# Allogeneic tumor cell-derived extracellular vesicles stimulate CD8 T cell response in colorectal cancer

**DOI:** 10.1016/j.omto.2023.100727

**Published:** 2023-09-16

**Authors:** Travis J. Gates, Dechen Wangmo, Xianda Zhao, Subbaya Subramanian

**Affiliations:** 1 Department of Pharmacology, University of Minnesota Medical School, Minneapolis, MN, USA; 2 Department of Surgery, University of Minnesota Medical School, Minneapolis, MN, USA; 3 Masonic Cancer Center, University of Minnesota Medical School, Minneapolis, MN, USA; 4 Center for Immunology, University of Minnesota Medical School, Minneapolis, MN, USA

**Keywords:** colorectal cancer, tumor extracellular vesicles, allogeneic, dendritic cells, T cells, immune checkpoint inhibitors

## Abstract

Most colorectal cancer (CRC) patients present with a microsatellite-stable phenotype, rendering them resistant to immune checkpoint inhibitors (ICIs). Among the contributors to ICI resistance, tumor-derived extracellular vesicles (TEVs) have emerged as critical players. Previously we demonstrated that autologous transfer of TEVs without miR-424 can induce tumor antigen-specific immune responses in CRC models. Therefore, we postulated that allogeneic TEVs, modified to lack miR-424 and derived from an MC38 cells, could induce CD8^+^ T cell responses while restraining CT26 cell-based tumor. Here, we show that prophylactic administration of MC38 TEVs, without miR-424, showed a significant augmentation in CD8^+^ T-cells within CT26 tumors. This allogenic TEV effect was evident in CT26 tumors but not B16-F10 melanoma. Furthermore, we demonstrated the capacity of dendritic cells (DCs) to internalize TEVs, a possible mechanism to elicit immune response. Our investigation of autologously administered DCs, which had been exposed to modified TEVs, underscores their potential to dampen tumor growth while elevating CD8^+^ T cell levels vis-a-vis MC38 wild-type TEVs exposed to DCs. Notably, the modified TEVs were well tolerated and did not increase peripheral blood cytokine levels. Our findings underscore the potential of modified allogeneic TEVs without immune-suppressive factors to elicit robust T cell responses and limit tumor growth.

## Graphical abstract

**Figure:**
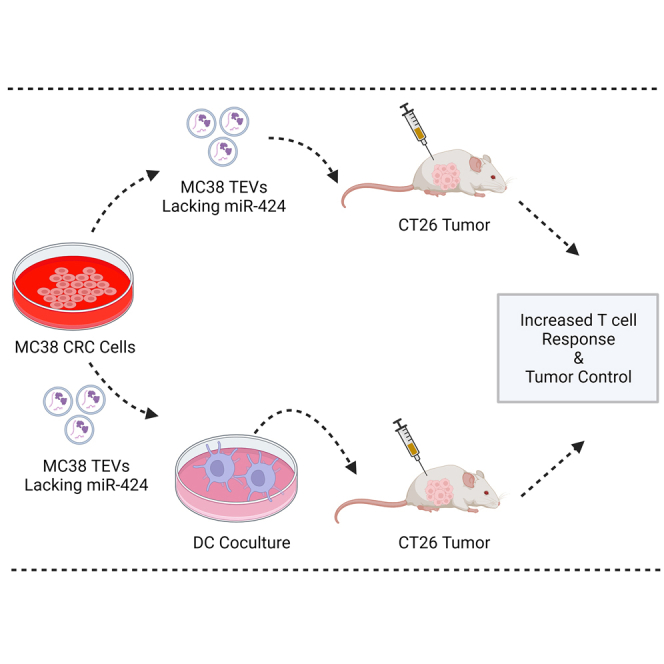

## Teaser

Subramanian and colleagues show that administration of MC38 CRC cell-derived tumor-derived extracellular vesicles (TEVs) lacking miR-424 boosted CD8^+^ T cell response in CT26 tumors and controlled tumor growth. These findings highlight the translational significance of allogeneic TEVs’ potential to trigger antitumor immune response and reduce tumor burden.

## Introduction

Colorectal cancer (CRC) is the third most prevalent cancer diagnosis in the United States and ranks as the second leading cause of cancer-related mortality. This scenario is further compounded by the escalating CRC incidence observed among patients below the age of 50 years.^1^ The emergence of immune checkpoint inhibitor (ICI) therapies, such as anti-PD1 and anti-CTLA4, have remarkably broadened therapeutic avenues across diverse malignancies.^2^^,^^3^^,^^4^ Nonetheless, the transformative potential of ICIs remains limited to a subset of CRC patients harboring the microsatellite instability-high (MSI-H) subtype, which constitutes less than 15% of the overall CRC population.^1^^,^^5^^,^^6^^,^^7^^,^^8^ Remarkably, most CRC patients, >85%, present with a microsatellite-stable (MSS) profile, rendering them unresponsive to ICIs.^6^^,^^9^ The immunogenic differences between the MSI and MSS phenotypes have emerged as a major predictive parameter governing the responsiveness to ICIs.^6^^,^^7^^,^^10^ The limited occurrence of immune cell infiltration within the subset of MSS-CRC cases further emphasizes the clinical urgency to decipher the intrinsic resistance mechanisms.^11^^,^^12^ Therefore, there is a critical clinical need to determine intrinsic resistance mechanisms and increase tumor T cell infiltration to synergize with ICIs.^13^^,^^14^

Interactions between tumor cells and immune cells within the tumor microenvironment (TME) are pivotal in shaping the effectiveness of CD8^+^ T cell-mediated anti-tumor immune responses and ICIs. The intricate mechanisms of tumor-immune cell crosstalk, facilitated by tumor-secreted extracellular vesicles (TEVs), have demonstrated their capacity to modulate the phenotype and function of immune cells within the TME.^15^^,^^16^ These TEVs can exert immunosuppressive or immunogenic effects contingent upon their cargo composition, a highly context-dependent phenomenon. Notably, the presence of PD-L1 in TEVs cargo has been implicated in local immunosuppression and subsequent depletion of PD-L1 from TEVs capable of reversing these suppressive effects has been observed.^17^^,^^18^

We demonstrated that syngeneic TEVs carrying microRNA miR-424, which targets CD28/CD80 costimulatory genes, act as contributors to immune suppression and promote resistance to ICIs in CRC.^19^ Conversely, TEVs with tumor antigens and other immunomodulatory factors like IFN-γ and dsDNA have demonstrated a potential to elicit immune responses.^20^^,^^21^^,^^22^ It is important to note that early studies examining the regulation of immune responses by TEVs were predominantly conducted within a syngeneic framework.^23^ However, translating modified TEVs from a syngeneic context into clinical implementation is challenging, largely stemming from inadequate tumor biomass to facilitate effective TEV isolation.^23^

Allogeneic EVs originating from dendritic cells (DCs) have demonstrated the capacity to elicit potent antitumor immune responses and enhance T cell memory, exhibiting potential therapeutic benefits.^24^ Previous studies have explored the stimulation of DCs with TEVs and other tumor antigens, yielding encouraging outcomes in preclinical models.^25^ However, the clinical efficacy of DC vaccine strategies remains confined to Sipuleucel-T in metastatic castration-resistant prostate cancer.^26^ Preclinical investigations into the utility of DC vaccines within the realm of CRC have yielded inconsistent results, further indicating the complex landscape of their effectiveness.^27^

The cargo composition of TEVs, including miR-424, has been postulated to generate local immune suppression, even in the presence of tumor antigens, primarily through inhibiting T cell costimulation.^19^ In this study, we determined the impact of allogeneic MC38 colon cancer TEVs without functional miR-424 (MC38-424i). Our findings reveal the substantial control in tumor growth through the prophylactic administration of MC38-424i TEVs in mice challenged with CT26 colon cancer cells but did not yield the same results in B16-F10 melanoma cells. Furthermore, our investigations underscore the feasibility of pulsing miR-424i TEVs onto DCs, which can be autologously transferred to BALB/c mice, resulting in augmented CD8^+^ T cell infiltration within tumors. Drawing insights from our studies, future endeavors could elucidate a potential synergistic interplay between allogeneic modified TEVs pulsed onto DCs and ICIs in the context of CRC.

## Results

### Allogeneic modified TEVs increase T cell infiltrates and decrease tumor growth

**Figure 1:**
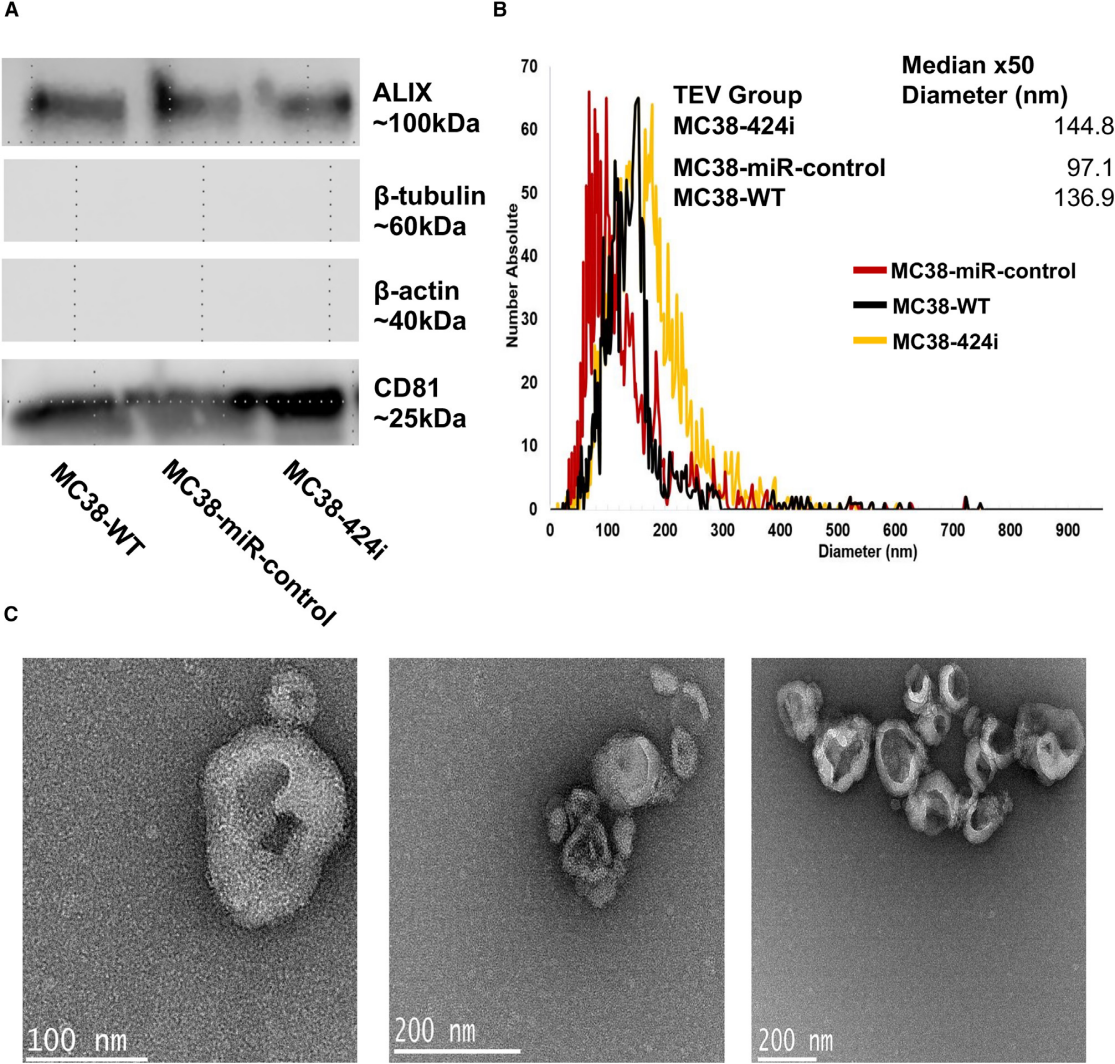
TEV isolation and characterization (A) Western blot data showing the expression of EV markers, including ALIX (∼100 kDa) and CD81 (∼25 kDa), alongside negative controls β-tubulin (∼60 kDa) and β-actin (∼40 kDa), for MC38 WT, MC38-miR-control, and MC38-424i TEVs. (B) Nanotracker analysis showcasing size distribution profiles of TEVs derived from MC38-424i (yellow), MC38-miR-control (red), and MC38-WT (black) TEVs. (C) Transmission electron microscopy images presenting the morphological characteristics of MC38-WT, MC38-miR-control, and MC38-424i TEVs.

Previously, we demonstrated that TEVs derived from CRC cells contain immunosuppressive miR-424, which impacts T cell costimulation and hinders the effectiveness of ICIs.^19^ We aimed to investigate the potential impact of allogeneic modified TEVs (TEVs characterized by the absence of functional miR-424) derived from MC38 colon cancer cell lines of a C57BL/6 background on BALB/c mice harboring CT26 cell-based tumors. To test this, we used MC38 wild-type (MC38-WT) cell lines and modified MC38 cell lines that stably express miR-424 inhibitor (MC38-424i) and MC38-424i scramble control (MC38 miR-control). First, we confirmed the quality of TEVs isolated from each cell line by western blotting and determined the EV markers CD81, and ALIX. We also included β-actin and β-tubulin to ensure no cellular contaminants were present in TEV isolation (Figure 1A). The presence of ALIX and CD81 was evident in MC38-WT, MC38-424i, and MC38-miR-control groups, while β-actin and β-tubulin were not observed. No positive signal was detected despite the potential for β-actin presence in TEV cytoskeletal components.

Subsequently, we conducted Nanotracker analysis to determine the size distribution across the MC38-WT, MC38-424i, and MC38-miR-control TEVs (Figure 1B). Notably, the average size distributions of TEVs were determined to be 136.9, 144.8, and 97.1 nm for MC38-WT, MC38-424i, and MC38-miR-control TEV groups, respectively. Transmission electron microscopy imaging gained further insight into TEV morphology, which provided additional validation of the TEV purity and structure (Figure 1C).

**Figure 2:**
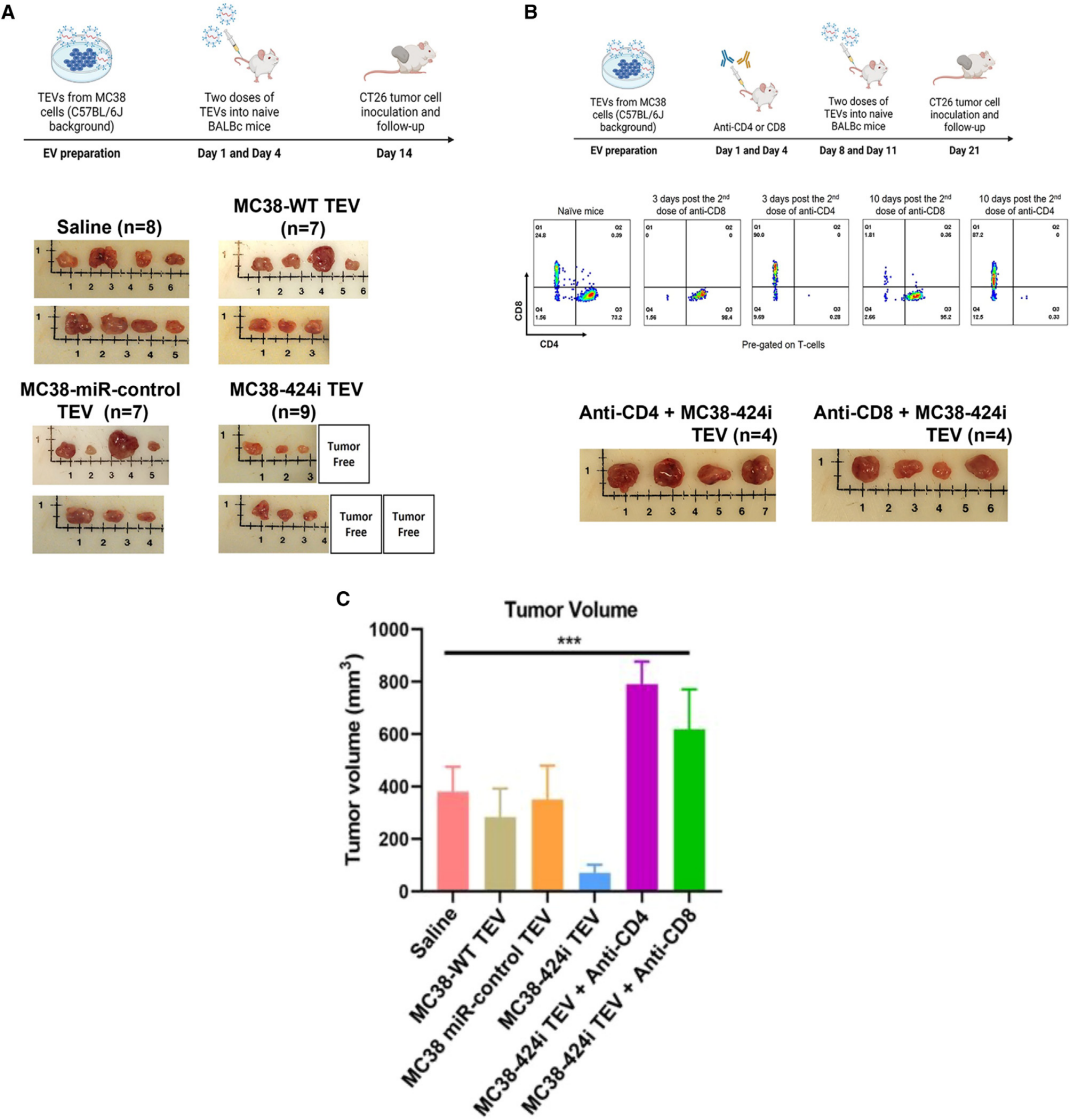
Treatment with allogeneic MC38 TEVs on CT26 tumor slows tumor progression (A) Schematic representation illustrating the administration of TEVs, tumor inoculation, and images capturing tumor progression within distinct groups (saline n = 8; MC38-WT TEV n = 7; MC38-miR-control TEV n = 7; MC38-424i TEV n = 9). (B) Flow cytometry validation confirming the depletion of α-CD4 and α-CD8 T cells, accompanied by tumor images post TEV inoculation in BALB/c mice bearing CT26 tumors. (C) Comparison of endpoint tumor volumes among different groups (saline, MC38-WT TEV, MC38-miR-control TEV, MC38-424i TEV, MC38-424i TEV + α-CD4, MC38-424i TEV + α-CD8, respectively). ∗∗∗p < 0.005; error bars ± SEM.

To investigate the potential impact of allogeneically modified TEVs on tumor growth, we prophylactically administered BALB/c mice with two injections of 10 μg each of MC38-WT, MC38-424i, MC38-miR-control TEVs, or saline, as illustrated in Figure 2A. A period of 10 days was allowed to develop an adaptive immune response, after which the mice were challenged with CT26 cells at a dose of 2 × 10^5^ cells per injection. After inoculation, a 21-day interval was permitted to allow for tumor progression. Mice treated with MC38-424i TEVs displayed strikingly smaller tumors (71.49 ± 30.32 mm^3^) in comparison with the saline group (380.9 ± 95.48 mm^3^), MC38-WT group (284.7 ± 108.1 mm^3^), and MC38-miR-control group (352.3 ± 128.5 mm^3^). Notably, within the MC38-424i group, three mice exhibited complete tumor regression following the CT26 tumor challenge, as shown in Figure 2A.

Furthermore, we sought to determine whether the observed tumor phenotype was contingent on the presence of CD4 or CD8 T cells. To ascertain this, we administered two intraperitoneal injections of depletion antibodies, targeting αCD4 and αCD8 (at a dose of 400 μg per injection), before administering allogeneic TEVs. The efficacy of CD4^+^ and CD8^+^ T cell depletion was evaluated using flow cytometry, comparing the depleted mice to naive spleen and lymph nodes 3 and 10 days after CD4^+^ and CD8^+^ depletion, as represented in Figure 2B. After implementing the experimental regimen outlined in Figure 2A, we confirmed that the depletion of CD4^+^ and CD8^+^ T cells in BALB/c mice compromised the influence of allogeneically modified TEVs on CT26 tumors. Specifically, MC38-424i and anti-CD4-treated mice exhibited augmented tumor volumes (791.5 ± 85.99 mm^3^), and MC38-424i and anti-CD8-treated mice displayed a similar trend (619.5 ± 151.5 mm^3^), as shown in Figure 2C. Therefore, we concluded that MC38-424i TEVs substantially inhibited tumor growth compared with MC38-WT, MC38-miR-control TEVs, and saline control groups. Furthermore, this observed phenotype appeared contingent on CD4^+^ and CD8^+^ T cells *in vivo*.

### Treatment with allogeneic modified TEVs increases tumor-infiltrating T cells

**Figure 3:**
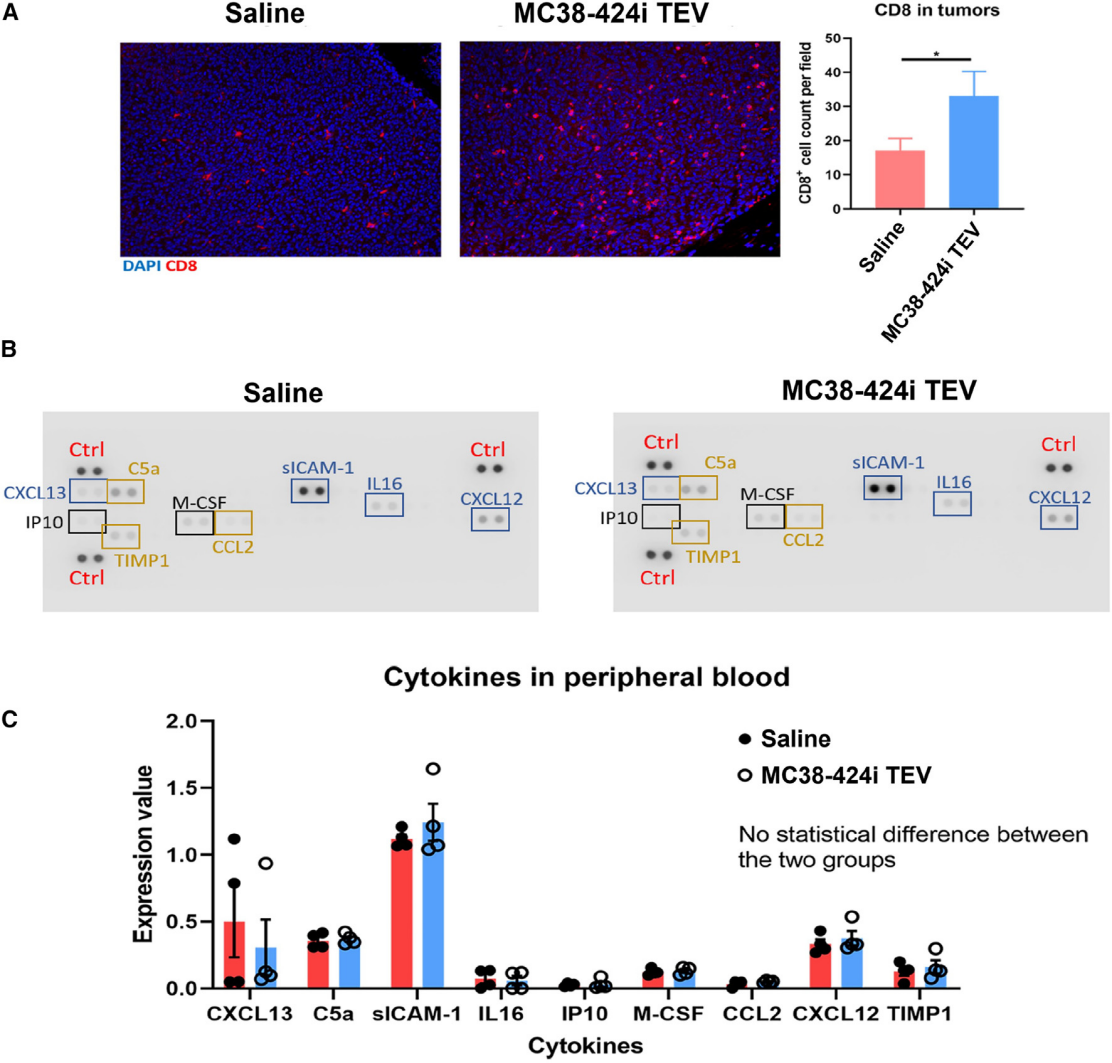
Treatment with allogeneic MC38 TEVs modulates T cell infiltrates (A) Immunofluorescence depicting CD8^+^ T cell distribution (red) and DAPI nuclear staining (blue) within CT26 tumors, comparing saline-treated (red) and MC38-424i TEV-treated (blue) groups. ∗p < 0.05; error bars ± SEM. (B) Peripheral blood cytokine levels from mice treated with saline or MC38-424i TEV. (C) Quantification of cytokine levels in peripheral blood, contrasting saline-treated (red) and MC38-424i TEV-treated (blue) groups. Error bars ± SEM.

Following the convincing observation of a substantial impact on tumor growth within the context of allogeneic MC38-424i TEVs in comparison with MC38-WT TEVs, MC38-miR-control TEVs, and the saline control groups, we proceeded with immunofluorescence analysis of tumor tissues. The objective was to determine the potential differences in T cell infiltration between CT26 tumors treated with allogeneic MC38-424i TEVs and those treated with saline. Our analysis revealed a significant difference in CD8^+^ cell counts per field, registering at 33.11 ± 7.21 and 17.17 ± 3.42 for allogeneic MC38-424i TEVs and saline treatments, respectively (Figure 3A).

Considering the broad-ranging influence of TEVs on cellular responses and the distinct genetic background of MC38-424i TEVs, we considered it key to investigate the safety profile of MC38-424i TEVs compared with saline. We assessed peripheral blood cytokines using a protein array, including tumor necrosis factor alpha (TNF-α), IL-6, IL-2, IL-10, and IFN-γ. Notably, our analysis indicated the absence of a significant increase in the detected cytokines between the allogeneic MC38-424i TEV and saline groups (Figure 3B).

### Allogeneic modified TEVs do not significantly influence B16-F10 tumor growth

**Figure 4:**
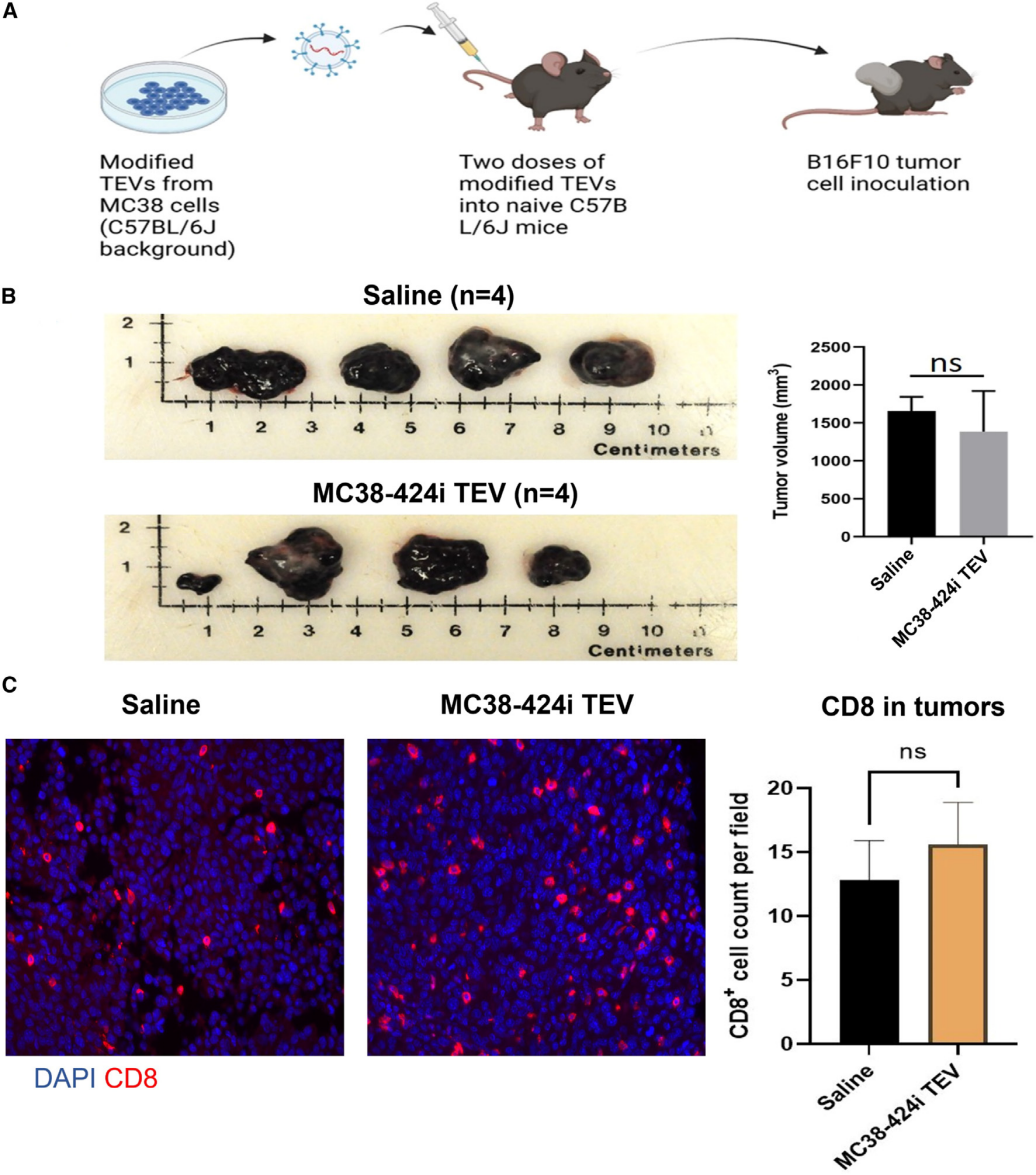
Treatment with MC38 TEVs on B16 melanoma tumors (A) Schematic representation of the administration of MC38 TEVs in C57BL/6J mice bearing B16-F10 tumors. (B) Comparison of tumor volumes between the saline-treated group (black) and the MC38-424i TEV-treated group (gray). ns, p > 0.05; error bars ± SEM. (C) Immunofluorescence visualization of CD8^+^ T cells (red) and DAPI nuclear staining (blue) within B16-F10 tumors, comparing the saline-treated group (black) and the MC38-424i TEV-treated group (orange). ns, p > 0.05; error bars ± SEM.

After observing a noticeable influence of MC38-424i TEVs on both CT26 colon cancer growth and T cell infiltration, we examined the specificity of this effect within the context of CRC tumor models. We adopted an analogous prophylactic model to investigate this, administering MC38-424i TEVs to C57BL/6 mice challenged with B16-F10 melanoma cells Figure 4A. Mice were administered two prophylactic doses (10 μg per injection) of allogeneic MC38-424i TEVs or saline on day 1 and day 4. On day 10, C57BL/6 mice were subjected to a subcutaneous injection containing 2 × 10^5^ B16-F10 melanoma cells. Remarkably, we did not observe a significant difference in endpoint tumor volumes, measuring 1,386 ± 536.1 and 1,657 ± 187.5 mm^3^ for the allogeneic modified TEV and saline groups, respectively (Figure 4B). However, substantial variability in tumor volume was evident within the allogeneic MC38-424i TEV group. In addition, analysis of CD8^+^ cell counts per field indicated no significant differences, revealing 12.8 ± 3.1 and 15.6 ± 3.3 for the allogeneic MC38-424i TEV and the saline groups, respectively (Figure 4C).

### Allogeneic TEV-pulsed DCs are instrumental in stimulating an anti-tumor immune response

**Figure 5:**
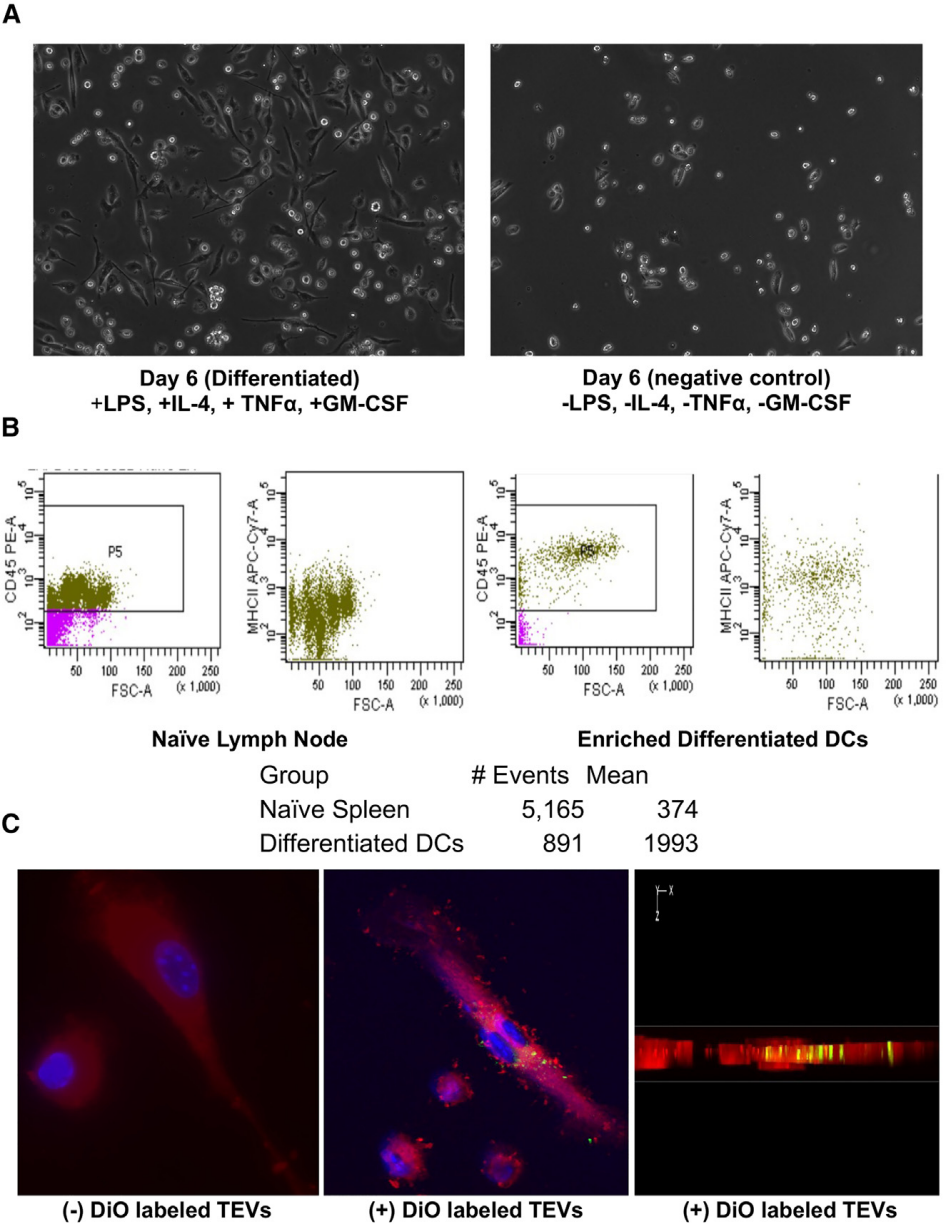
Dendritic cell isolation and TEV capture *in vitro* (A) Comparison of DC morphology on day 6 with (left) and without (right) differentiation induced by Il-4, TNF-α, GM-CSF, and LPS. (B) Mean fluorescence intensity of MHC class II-APC-Cy7 in naive splenocytes and day 6 *in vitro* differentiated DCs. (C) Fluorescence microscopy images demonstrating the uptake of TEVs (red) by DCs, with and without labeling of TEVs using DiO (green). Nuclei stained with DAPI are shown in blue. A cross-sectional view (X-Z plane) of z stack images containing DiO-labeled TEVs is also presented.

Upon observing the tumor-specific activation of the immune response induced by allogeneic modified TEVs, we investigated the mechanistic underpinnings of TEV processing in an *in vitro* setting. We hypothesized that DCs capture and present TEVs, thereby enabling the exhibition of tumor antigens within these TEVs to T cells. This presentation could consequently elicit an anti-tumor immune response. Such a phenomenon could also explain the observed indirect detriment to the phenotype upon depletion of CD4^+^ and CD8^+^ T cells. To assess the plausibility of TEV capture by DCs, we isolated monocytes and fostered *in vitro* differentiation into DCs by introducing Il-4, GM-CSF, TNF-α, and LPS. Subsequently, we performed imaging of the DC populations over 6 days, both in the presence and absence of differentiation factors (Figure 5A).

Furthermore, we conducted flow cytometric analysis, confirming the expression of MHC class II between enriched DCs (mean signal intensity of 1,993) and undifferentiated monocytes sourced from naive spleen and lymph nodes (mean signal intensity of 374) (Figure 5B). Subsequently, we carried out an *in vitro* experiment designed to assess the uptake of TEVs. TEVs were stained with the lipophilic dye DiO and introduced to DCs cultured on fibronectin-coated glass slides (5 μg/mL). Following a 24-h incubation period, we proceeded to visualize the outcome. Remarkably, we observed the intracellular localization of the DiO signal (green) within the X-Z plane of the DCs that had been stained with Cytopainter Red (Figure 5C).

**Figure 6:**
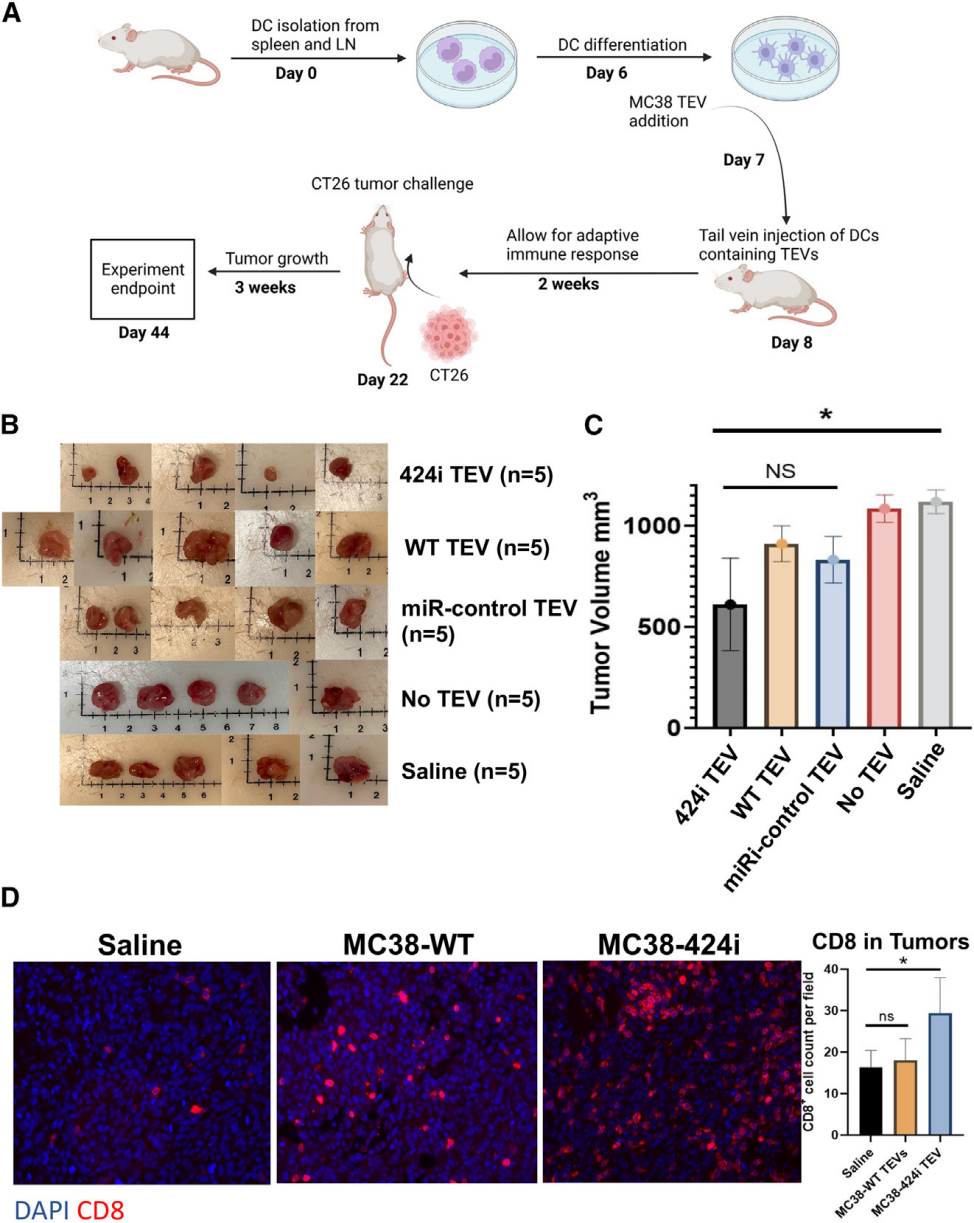
Autologous transfer of DCs exposed to TEVs slows tumor growth (A) Illustration depicting the process of DC isolation, TEV pulsing, and autologous transfer of DCs to BALB/c animals before tumor challenge with CT26 colon cancer cells. (B) Images showing the tumor status at the experimental endpoint for the MC38-424i, MC38 WT, MC38-miR-control, No TEV, and saline groups (n = 5/group). (C) Graph depicting the tumor volumes at the experimental endpoint for the MC38-424i, MC38-WT, MC38-miR-control, no TEV, and saline groups (n = 5/group). ∗p < 0.05; ns, p > 0.05; error bars ± SEM. (D) Immunofluorescence images and quantification of CD8+ T cells (red) and DAPI-stained nuclei (blue) in CT26 tumors among the saline, MC38-WT, and MC38-424i TEV treatment groups. ∗p < 0.05; ns, p > 0.05; error bars ± SEM.

Having established that DCs can capture MC38 TEVs, we examined the potential of allogeneic MC38-424i TEVs pulsed onto DCs derived from a BALB/c background in cultured conditions to confer protection against CT26 tumor challenge. This approach could enable the targeted delivery of TEVs without necessitating their direct administration into the bloodstream. To evaluate this strategy, we enriched DCs from the spleen of BALB/c mice and subjected them to MC38-WT, MC38-miR-control, and MC38-424i TEVs on the sixth day of DC differentiation. The following day, we autologously transferred 1 × 10^6^ DCs exposed to TEVs via intravenous tail vein injection. Our experimental design encompassed five groups of BALB/c mice (n = 5/group): MC38 WT TEV, MC38-424i TEV, MC38-miR-control TEV, no TEV, and saline. After allowing a 14-day interval following DC administration, we introduced a CT26 tumor challenge, allowing tumor progression over 21 days (Figure 6A).

Substantial variations in tumor volumes were evident when comparing all TEV groups with both DCs without TEVs (1,105 ± 37.4 mm^3^) and saline (1,186.4 ± 25.2 mm^3^) groups, showing a significant difference. Intriguingly, no notable differences in tumor volumes were observed among the MC38-WT TEV (834.4 ± 65.4 mm^3^), MC38-miR-control TEV (910.6 ± 84.4 mm^3^), and MC38-424i TEV (655.8 ± 355.4 mm^3^) groups (Figures 6B and 6C). Notably, MC38-424i TEVs (655.8 ± 355.4 mm^3^) revealed a significant difference in tumor volumes compared with the no TEVs (1,105 ± 37.4 mm^3^) and saline groups (1,186.4 ± 25.2 mm^3^).

Despite the absence of noticeable differences in tumor volumes across the TEV groups, we investigated CD8^+^ T cell infiltrates. Remarkably, we observed significant variations in CD8^+^ cell counts per field. We detected 29.4 ± 8.6, 18.0 ± 5.2, and 16.4 ± 4.0 for the MC38-424i TEV, MC38 WT TEV, and saline groups, respectively (Figure 6D).

## Discussion

This study focused on understanding the consequences of administering MC38 allogeneic TEVs, which lack functional miR-424, on tumor growth dynamics and T cell infiltrates in mice harboring CT26 or B16-F10 tumors. We hypothesized that the administration of MC38-424i TEVs would control the growth of both CT26 and B16-F10 tumors.

Substantiating this hypothesis, we observed a substantial reduction in tumor volumes upon the prophylactic administration of MC38-424i TEVs to mice subjected to CT26 tumor challenges (Figures 2A and 2C). However, in the context of C57BL/6 animals challenged with B16-F10 tumors, we did not observe any differences in endpoint tumor volumes following the administration of MC38-424i TEVs (Figure 4B). Despite this, two animals displayed diminished tumor sizes at the experimental endpoint; nevertheless, the group variability did not yield statistically significant outcomes.

It has been reported that conserved tumor exon junctions between MC38 cells and B16-F10 cells can be presented on MHC class I.^28^ Furthermore, prophylactic exposure to these conserved tumor exon junctions has been reported to confer protective effects against B16-F10 growth, attributed to elicited anti-tumor immune responses.^28^ Given these findings, we speculate that conserved tumor neoantigens present within MC38 TEVs and B16-F10 melanoma cells might undergo immunoediting, ultimately facilitating immune evasion.^29^^,^^30^

On the contrary, the tumor neoantigens shared between MC38 TEVs and B16-F10 tumors might evoke a comparatively weak immune response.^31^ Notably, existing literature highlights that MC38 cells and CT26 cell lines bear a comparable load of somatic mutations per megabase, resulting in a similar tumor mutational burden.^32^ These two observations collectively explain the divergent outcomes observed in our study. Specifically, the robust immune response (Figure 3A) and the conferred protective effects on tumor growth (Figure 2C) witnessed with MC38-424i TEVs in CT26 tumors can be attributed to these factors, whereas a parallel immune response was not attained in B16-F10 tumors. Similarly, we documented a substantial augmentation in CD8^+^ T cells within CT26 tumors following miR-424i TEV treatment (Figure 3A), whereas no corresponding increment was discerned in CD8^+^ T cells within B16-F10 tumors receiving the same treatment (Figure 4C). These observations underscore that the origin of tissue or similarities in tumor mutational burden could play a pivotal role in determining the efficacy of allogeneic TEVs. This is substantiated by the observation that the impact of MC38-424i TEVs on B16-F10 immune infiltrates and tumor growth was relatively subdued.

Furthermore, it is noteworthy that no significant increase in peripheral blood cytokines was observed upon comparing allogeneic MC38-424i TEVs with saline. These data support that the administration of TEVs was well tolerated within an allogeneic setting (Figure 3B). This observation is significant, as any induction of a cytokine release syndrome profile could undermine the clinical translatability of allogeneic TEVs.^33^^,^^34^

In addition, our investigation aimed to establish the capacity of DCs to capture TEVs and whether *in-vitro*-differentiated DCs could be pulsed with allogeneic MC38 TEVs, subsequently enabling their autologous transfer back into BALB/c mice to initiate an anti-tumor immune response in CT26 tumors. This paradigm enables the delivery of autologous tumor antigens without directly administering TEVs into the bloodstream. Convincing evidence emphasizes the role of antigen-presenting cells (APCs), such as DCs, as potent orchestrators of efficacious anti-tumor responses by presenting antigens to T cells.^35^ Previous studies have explored the exposure of tumor cells and antigens to DCs (exemplified by Sipuleucel-T) and revealed varied levels of efficacy in the context of prostate cancer.^26^^,^^36^^,^^37^ In this context, we postulated that the abundance of endogenous TEVs harboring miR-424, as demonstrated in our prior investigation,^19^ might still impede the effective translation of DC vaccine strategies into clinical applications. Our data suggest that modified allogeneic TEVs can be successfully pulsed onto DCs *in vitro* (Figure 5C). Additional images confirming TEV uptake can be referenced in Figure S1.

Moreover, the autologous transfer of DCs to BALB/c mice demonstrated their potential in conferring protection against tumor growth, as evidenced by comparisons with the no TEVs and saline groups (Figures 6A–6C). While noticeable differences in tumor volumes within the allogeneic MC38 TEV groups were not observed, a striking contrast emerged regarding CD8^+^ T cell infiltration. Specifically, DCs pulsed with allogeneic MC38-424i TEVs showed a significant distinction in CD8^+^ T cell infiltration compared with MC38-WT TEV. Our findings hold significant implications in the context of advanced tumors, which are often intrinsically immunosuppressive and unresponsive to ICIs due to complex mechanisms limiting functional CD8^+^ T cell infiltration.^38^^,^^39^ Our data signify the potential of DCs loaded with allogeneic MC38-424i TEVs in promoting CD8^+^ T cell infiltration into tumors (Figure 6D).

An additional component we explored was the capacity of MC38-424i TEVs, when loaded onto DCs, to elicit anti-tumor immune responses, thereby circumventing the direct administration of TEVs into the bloodstream of BALB/c mice (Figure 5C). Given the promising outcomes, it would be worthwhile for future investigations to incorporate the autologous transfer of DCs pulsed with MC38-424i TEVs. This endeavor would aim to ascertain whether ICI efficacy in CRC preclinical models could be influenced before the clinical translation phase.

Although our study reveals differences in tumor growth and immune response between CT26 and B16-F10 tumors following MC38-424i TEV administration, the underlying mechanisms responsible for these disparities are not fully elucidated. Further investigations addressing the potential molecular, cellular, and microenvironmental factors influencing these contrasting responses are essential to provide a comprehensive understanding. Furthermore, while the study successfully demonstrates the loading of allogeneic MC38-424i TEVs onto DCs *in vitro* and their subsequent impact on tumor growth and immune response *in vivo*, the translational relevance of these findings to clinical scenarios remains a limitation. Directly extrapolating the results to humanized models and clinical applications requires careful consideration of inter-species variations and the complex immune interactions in human systems.

Moreover, our study predominantly centers on CD8^+^ T cell infiltration and tumor growth, with limited exploration of other immune components that could contribute to the observed outcomes. A broader profiling of the immune landscape, encompassing various immune cell subsets, cytokine profiles, and immunosuppressive factors, would provide a more comprehensive understanding of the immune dynamics influenced by allogeneic TEVs. Addressing these limitations through further research and expanding the investigation scope could enhance the findings' robustness and clinical relevance.

In conclusion, this study underscores the protective potential of prophylactically administered allogeneic MC38 TEVs, lacking functional miR-424, against CT26 tumor growth facilitated by eliciting anti-tumor immune responses. Our investigations also reveal the tolerability of allogeneic MC38-424i TEVs, as they did not trigger significant changes in peripheral blood cytokine expression. Moreover, we established the feasibility of loading allogeneic MC38 TEVs onto DCs. Notably, the autologous transfer of modified TEV-pulsed DCs to mice demonstrated protective effects against CT26 tumor challenges, resulting in elevated CD8^+^ T cell infiltrates compared with MC38-WT TEVs and saline.

These findings warrant further investigation into the synergy between modified TEV-pulsed DCs and ICIs with orthotopic preclinical models of CRC. Deciphering the response to ICI treatment in the presence of DCs loaded with allogeneic TEVs demands a comprehensive and advanced preclinical investigation. Experiments integrating a repertoire of immunotherapies designed to activate DCs in conjunction with ICIs are prerequisites before the translation and clinical implementation phases.

## Materials and methods

### Mice and animal husbandry

All animal studies were approved by the Institutional Animal Care and Use Committee (IACUC) of the University of Minnesota. All mice were housed in specific pathogen-free conditions with fully autoclaved cages to minimize non-tumor-specific immune activation. BALB/c and C57BL/6 mice were purchased from The Jackson Laboratory. Mice were bred in-house and were used for experiments between age 6 and 8 weeks.

### Cell lines and cell culture

Mouse CRC cell line CT26 (ATCC CRL-2638) was purchased from ATCC. Dr. Nicholas Haining kindly provided mouse CRC cell line MC38. MC38 cells stably expressing miR322 inhibitor mouse homolog to miR-424 (MC38-424i) and MC38-miR-control cells were used in this study as described by Zhao et al.^19^ CT26 cells were cultured in complete Roswell Park Memorial Institute Medium (RPMI) 1640 (Gibco), supplemented with 10% heat-inactivated fetal bovine serum (FBS) (Thermo Fisher Scientific), 100 IU/mL penicillin, and 100 μg/mL streptomycin (Invitrogen Life Technologies). MC38-WT, MC38-424i, and MC38-miR-control cells were cultured in the complete Dulbecco’s modified Eagle’s medium (DMEM) (Gibco), supplemented with 10% heat-inactivated FBS, 100 IU/mL penicillin, and 100 μg/mL streptomycin. B16-F10 melanoma cells (ATCC CRL-6475) were purchased from ATCC and were cultured in the complete DMEM (Gibco), supplemented with 10% heat-inactivated FBS, 100 IU/mL penicillin, and 100 μg/mL streptomycin. Cell lines were authenticated and routinely tested for mycoplasma.

### Isolation of TEVs

Twenty-four hours before TEV isolation, cell medium was changed to DMEM supplemented with 10% exosome-depleted FBS, 100 IU/mL penicillin, and 100 μg/mL streptomycin. A standardized differential centrifugation protocol was used to purify TEVs from cell culture supernatants from MC38-WT, MC38-424i, and MC38-miR-control cells. Cell culture supernatants were centrifuged at 300 × *g* for 10 min to remove cells. Supernatants were centrifuged for 3,000 × *g* for 10 min to remove dead cells. Supernatants were centrifuged at 10,000 × *g* for 30 min to remove cellular debris. Supernatants were centrifuged at 2,000 × *g* for 30 min in Amicon Ultra-15 (Millipore) to concentrate supernatant. Supernatants were ultracentrifuged at 100,000 × *g* for 70 min at 4°C with a SW40Ti rotor (Beckman Coulter). Pelleted TEVs were suspended and washed in PBS and underwent ultracentrifugation at 100,000 × *g* for 70 min at 4°C. TEVs were collected in PBS for downstream analysis and experimentation.

### Characterization of TEVs

To characterize the purified TEVs, we first used electron microscopy. TEVs suspended in PBS were placed on Formvar carbon-coated nickel grids. TEVs on grids were stained with 2% uranyl acetate and allowed to air dry. TEVs were visualized using an FEI Tecnai G2 F30 Field Emission Gun Transmission Electron Microscope with a 4k × 4k ultrascan charge-coupled device camera. The size distributions and concentration of TEVs isolated from cell culture supernatants were determined using the NanoSight LM-10 microscope (Malvern Instruments) equipped with particle tracking software. Ten independent microscopic fields were captured and analyzed per cell line sample. Data were merged and presented as a single histogram plot. In addition, we tested TEV-related protein markers from isolated TEVs using western blotting. TEV protein concentration was estimated by using the Pierce Micro BCA Protein Assay Kit. Ten micrograms of TEV protein from MC38 and modified cell lines were loaded on SDS gels. Primary antibodies binding markers: CD81 (1:1,000, BioLegend, cat. no. 104902) and ALIX (1:2,000, BioLegend, cat. no. 634502) were used to validate TEV protein markers. β-Actin (1:1,500, Cell Signaling, cat. no. 8H10D10) and β-tubulin (1:2,000, Invitrogen, cat. no. MA5 16308) confirmed no cellular contaminates.

### Prophylactically modified MC38 TEV administration in CT26 and B16-F10 subcutaneous tumor models

Ten micrograms of MC38-WT, MC38-424i, and MC38 miR-control TEVs suspended in sterile saline control were prophylactically injected twice into the tail vein of BALB/c or C57BL/6 mice, depending on the experimental setup. We allowed 2 weeks for an adaptive immune response before CT26 or B16-F10 tumor cell challenge. To establish subcutaneous tumors, we injected 2 × 10^5^ CT26 colon cancer cells suspended in 100 μL of 50:50 RPMI:Matrigel (Corning) into the right flank of BALB/c mice. Furthermore, 2 × 10^5^ B16-F10 melanoma cells were injected in the same preparation into the right flank of C57BL/6 mice. After tumor cell inoculation, tumors were measured 3 times per week using an electronic caliper. Tumor volumes were calculated using the formula (volume = [width^2^ × length]/2). Mice were sacrificed at 21 days following tumor inoculation. Tumor tissues were excised, imaged, and fixed in 10% neutral buffered formalin overnight. Fixed tissues were paraffin-embedded and sectioned in the University of Minnesota Clinical and Translational Sciences Institute. Mouse peripheral cytokines were measured using the Mouse Proteome Profiler Kit (R&D Systems) following the manufacturer’s protocol on 100 μL of mouse serum from whole blood.

### Immunofluorescence of tumor tissues

Formalin-fixed paraffin-embedded tissues were deparaffinized with three xylene washes, rehydrated with gradient ethanol, and underwent antigen retrieval in antigen retrieval buffer (AR9, PerkinElmer) in a 95°C water bath. Sections were blocked in 5% bovine serum albumin buffer for 30 min. Primary anti-mouse antibody CD8 (1:100, Abcam, cat. no. ab217344) was added and incubated overnight at 4°C. The tissue sections were washed twice with PBS and incubated with secondary antibodies (goat-anti-rat-A568, 1:250, Invitrogen, cat. no. A11077) and (goat-anti-rabbit-A568, 1:250, Invitrogen, cat. no. A11011) for 1 h. Tissues were washed twice with PBS, and slides were mounted with slide mounting medium with DAPI (Abcam, cat. no. ab104139). Slides were imaged on the BZX810 fluorescence microscope (Keyence). Quantitative image analysis was performed by counting positive signal percentage and fluorescence intensity signal in at least five randomly selected fields of each tumor tissue core.

### CD4^+^ and CD8^+^ T cell depletion

T cell subsets were depleted by intraperitoneally administering 400 μg of depleting antibody twice before prophylactic administration of MC38 TEVs and CT26 tumor challenge. CD4 T cells were depleted with anti-CD4 mAb (Clone GK1.5, Bio X Cell). CD8 T cells were depleted with anti-CD8α (Clone 2.43, Bio X Cell). CD4 and CD8 T cell depletion were confirmed using flow cytometry on the BD FACS CantoII (BD Biosciences) from the mouse spleen, lymph node, and peripheral blood. Antibodies for flow cytometry were CD3-APC (1:200, BioLegend, cat. no. 100236), CD8-APC-Cy7 (1:100, BioLegend, cat. no. 100714), CD4-BV510 (1:100, BioLegend, cat. no. 100449), CD11b-FITC (1:100, BioLegend, cat. no. 101206), CD19-FITC (1:100, BioLegend, cat. no. 115506), Nk1.1-FITC (1:100, BioLegend, cat. no. 108706), and CD45-PE (1:200, BioLegend, cat. no.103106).

### Monocyte isolation and DC differentiation

According to the manufacturer’s protocol, the monocytes were isolated using a negative selection from the mouse spleen, inguinal, axillary, and brachial lymph nodes with the Dynabeads Mouse DC Enrichment Kit (Invitrogen, cat. no. 11429D). Monocytes were plated at 10^7^ cells/well and differentiated using a Dendritic Cell Differentiation Kit (R&D Systems, cat. no. CDK004) according to the manufacturer’s instructions. DCs were differentiated for 6 days in the kit’s medium supplemented with 250 IU/mL IL-4 and 800 IU/mL granulocyte macrophage colony-stimulating factor for 2 days. Then the cells were centrifuged and cultured in fresh complete medium supplemented with 2,000 IU/mL IL-4 and 2,000 IU/mL granulocyte macrophage colony-stimulating factor. On day 6, cells were centrifuged and resuspended in medium supplemented with 2,000 IU/mL IL-6, 400 IU/mL IL-1β, 2,000 IU/mL TNF-α, and 100 ng/mL lipopolysaccharide, and were cultured for a further 24 h. On day 7, we confirmed DC differentiation with flow cytometry to see the percentage of cells expressing a high level of MHC class II. Antibodies in flow cytometry were CD3-FITC (1:100, BioLegend, cat. no.100204), CD19-FITC (1:100, BioLegend, cat. no.115506), Nk1.1-FITC (1:100 BioLegend, cat. no. 108706), CD45-PE (1:200, BioLegend, cat. no. 103106), CD11b-APC (1:100, BioLegend, cat. no. 101212), IA/12E-APC-Cy7 (1:100, BioLegend, cat. no. 107628), and CD11c-BV510 (1:100, BioLegend, cat. no. 117338).

### DC TEV uptake experiment

DCs and TEVs were isolated as previously described. DCs were plated on Labtek II chambers with chamber protectors coated with fibronectin (5 μg/mL) and differentiated in the same manner as previously described. TEVs from MC38 cell lines were stained with DiO lipophilic dye (Invitrogen) for 30 min and washed three times with PBS and ultracentrifugation at 100,000 × *g* for 70 min at 4°C. TEV pellets labeled with DiO were suspended in DC medium, and 1 μg TEVs were added to DC cultures on day 6 with 100 ng/mL LPS. Following 24 h to allow for TEV uptake, DC culture on glass slides was stained with Cytopainter Red (Abcam, cat. no. ab219942) for 30 min according to the manufacturer’s instructions and fixed with 4% formaldehyde for 30 min and washed 5 times with PBS. Chamber protectors were removed, slides were mounted with mounting medium with DAPI, and DCs exposed to MC38 TEVs were imaged on the BZX810 fluorescence microscope (Keyence). A series of photos were taken with a (4.4 μm) stepwise increase (0.4 μm) at each step on the z axis to validate the intracellular uptake of TEVs prior to the *in vivo* experiment.

### Prophylactic autologous TEV DC animal model

DCs were isolated and differentiated as previously described from BALB/c mice. TEVs from MC38-WT, MC38 322i, and MC38 miRi-control were isolated as described previously. On day 6, 10 μg of TEVs from the MC38 cell lines were administered to DCs in culture. On day 7, DCs were scraped from 10 cm^3^ dishes and counted and dosed at 1 × 10^6^ cells/injection in 100 μL of sterile PBS. Five groups (n = 5/group) of animals were injected prophylactically with intravenous tail vein injection of autologous DC cell suspensions of (MC38-WT-DCs, MC38 322i-DCs, MC38 miRi-control-DCs, No TEV control-DCs, and saline. BALB/c mice underwent 2 × 10^5^ CT26 subcutaneous tumor challenge 14 days following the administration of DCs. Tumors were measured 3 times a week for 21 days using an electronic caliper. Mice were sacrificed at 21 days following tumor inoculation. Tumor tissues were excised, imaged, and fixed in 10% neutral buffered formalin. Fixed tissues were paraffin embedded and sectioned in the University of Minnesota Clinical and Translational Sciences Institute.

### Statistics

We used GraphPad Prism, versions 6.0 and 8.0, to perform statistical analyses and visualize data. We used the Student’s t test to compare the treatment and control arms. One-way ANOVA was used when comparing more than two groups. All data are plotted as the mean ± standard error of the mean (SEM). All statistics were evaluated at two-tailed α = 0.05 unless otherwise corrected for multiple comparisons.

## Data and code availability

No large datasets were generated or analyzed in this study.

## Supplemental information

Document S1. Figure S1

Document S2. Article plus supplemental information

## References

[1] Siegel R.L., Miller K.D., Wagle N.S., Jemal A. (2023). Cancer statistics, 2023. CA. Cancer J. Clin..

[2] Larkin J., Chiarion-Sileni V., Gonzalez R., Grob J.J., Rutkowski P., Lao C.D., Cowey C.L., Schadendorf D., Wagstaff J., Dummer R. (2019). Five-Year Survival with Combined Nivolumab and Ipilimumab in Advanced Melanoma. N. Engl. J. Med..

[3] Motzer R.J., Escudier B., McDermott D.F., George S., Hammers H.J., Srinivas S., Tykodi S.S., Sosman J.A., Procopio G., Plimack E.R. (2015). Nivolumab versus Everolimus in Advanced Renal-Cell Carcinoma. N. Engl. J. Med..

[4] Hellmann M.D., Paz-Ares L., Bernabe Caro R., Zurawski B., Kim S.W., Carcereny Costa E., Park K., Alexandru A., Lupinacci L., de la Mora Jimenez E. (2019). Nivolumab plus Ipilimumab in Advanced Non–Small-Cell Lung Cancer. N. Engl. J. Med..

[5] Le D.T., Uram J.N., Wang H., Bartlett B.R., Kemberling H., Eyring A.D., Skora A.D., Luber B.S., Azad N.S., Laheru D. (2015). PD-1 Blockade in Tumors with Mismatch-Repair Deficiency. N. Engl. J. Med..

[6] Le D.T., Durham J.N., Smith K.N., Wang H., Bartlett B.R., Aulakh L.K., Lu S., Kemberling H., Wilt C., Luber B.S. (2017). Mismatch repair deficiency predicts response of solid tumors to PD-1 blockade. Science.

[7] André T., Shiu K.-K., Kim T.W., Jensen B.V., Jensen L.H., Punt C., Smith D., Garcia-Carbonero R., Benavides M., Gibbs P. (2020). Pembrolizumab in Microsatellite-Instability–High Advanced Colorectal Cancer. N. Engl. J. Med..

[8] Boland C.R., Goel A. (2010). Microsatellite instability in colorectal cancer. Gastroenterology.

[9] Mandal R., Samstein R.M., Lee K.W., Havel J.J., Wang H., Krishna C., Sabio E.Y., Makarov V., Kuo F., Blecua P. (2019). Genetic diversity of tumors with mismatch repair deficiency influences anti-PD-1 immunotherapy response. Science.

[10] Cercek A., Lumish M., Sinopoli J., Weiss J., Shia J., Lamendola-Essel M., El-Dika I.H., Segal N., Shcherba M., Sugarman R. (2022). PD-1 Blockade in Mismatch Repair–Deficient, Locally Advanced Rectal Cancer. N. Engl. J. Med..

[11] Pagès F., Mlecnik B., Marliot F., Bindea G., Ou F.S., Bifulco C., Lugli A., Zlobec I., Rau T.T., Berger M.D. (2018). International validation of the consensus Immunoscore for the classification of colon cancer: a prognostic and accuracy study. The Lancet.

[12] Angell H.K., Bruni D., Barrett J.C., Herbst R., Galon J. (2020). The Immunoscore: Colon Cancer and Beyond. Clin. Cancer Res..

[13] Sinicrope F.A. (2022). Increasing Incidence of Early-Onset Colorectal Cancer. N. Engl. J. Med..

[14] Akimoto N., Ugai T., Zhong R., Hamada T., Fujiyoshi K., Giannakis M., Wu K., Cao Y., Ng K., Ogino S. (2021). Rising incidence of early-onset colorectal cancer - a call to action. Nat. Rev. Clin. Oncol..

[15] Kalluri R., LeBleu V.S. (2020). The biology, function, and biomedical applications of exosomes. Science.

[16] Prakash A., Gates T., Zhao X., Wangmo D., Subramanian S. (2023). Tumor-derived extracellular vesicles in the colorectal cancer immune environment and immunotherapy. Pharmacol. Ther..

[17] Chen G., Huang A.C., Zhang W., Zhang G., Wu M., Xu W., Yu Z., Yang J., Wang B., Sun H. (2018). Exosomal PD-L1 contributes to immunosuppression and is associated with anti-PD-1 response. Nature.

[18] Poggio M., Hu T., Pai C.C., Chu B., Belair C.D., Chang A., Montabana E., Lang U.E., Fu Q., Fong L., Blelloch R. (2019). Suppression of Exosomal PD-L1 Induces Systemic Anti-tumor Immunity and Memory. Cell.

[19] Zhao X., Yuan C., Wangmo D., Subramanian S. (2021). Tumor-Secreted Extracellular Vesicles Regulate T-Cell Costimulation and Can Be Manipulated To Induce Tumor-Specific T-Cell Responses. Gastroenterology.

[20] Horrevorts S.K., Stolk D.A., van de Ven R., Hulst M., van Het Hof B., Duinkerken S., Heineke M.H., Ma W., Dusoswa S.A., Nieuwland R. (2019). Glycan-Modified Apoptotic Melanoma-Derived Extracellular Vesicles as Antigen Source for Anti-Tumor Vaccination. Cancers.

[21] Wolfers J., Lozier A., Raposo G., Regnault A., Théry C., Masurier C., Flament C., Pouzieux S., Faure F., Tursz T. (2001). Tumor-derived exosomes are a source of shared tumor rejection antigens for CTL cross-priming. Nat. Med..

[22] Diamond J.M., Vanpouille-Box C., Spada S., Rudqvist N.P., Chapman J.R., Ueberheide B.M., Pilones K.A., Sarfraz Y., Formenti S.C., Demaria S. (2018). Exosomes Shuttle TREX1-Sensitive IFN-Stimulatory dsDNA from Irradiated Cancer Cells to DCs. Cancer Immunol. Res..

[23] Samuel M., Gabrielsson S. (2021). Personalized medicine and back–allogeneic exosomes for cancer immunotherapy. J. Intern. Med..

[24] Larssen P., Veerman R.E., Akpinar G.G., Hiltbrunner S., Karlsson M.C.I., Gabrielsson S. (2019). Allogenicity Boosts Extracellular Vesicle-Induced Antigen-Specific Immunity and Mediates Tumor Protection and Long-Term Memory In Vivo. J. Immunol..

[25] Goldszmid R.S., Idoyaga J., Bravo A.I., Steinman R., Mordoh J., Wainstok R. (2003). Dendritic cells charged with apoptotic tumor cells induce long-lived protective CD4+ and CD8+ T cell immunity against B16 melanoma. J. Immunol..

[26] Kantoff P.W., Higano C.S., Shore N.D., Berger E.R., Small E.J., Penson D.F., Redfern C.H., Ferrari A.C., Dreicer R., Sims R.B. (2010). Sipuleucel-T Immunotherapy for Castration-Resistant Prostate Cancer. N. Engl. J. Med..

[27] Wooster A.L., Girgis L.H., Brazeale H., Anderson T.S., Wood L.M., Lowe D.B. (2021). Dendritic cell vaccine therapy for colorectal cancer. Pharmacol. Res..

[28] Burbage M., Rocañín-Arjó A., Baudon B., Arribas Y.A., Merlotti A., Rookhuizen D.C., Heurtebise-Chrétien S., Ye M., Houy A., Burgdorf N. (2023). Epigenetically controlled tumor antigens derived from splice junctions between exons and transposable elements. Sci. Immunol..

[29] Dunn G.P., Bruce A.T., Ikeda H., Old L.J., Schreiber R.D. (2002). Cancer immunoediting: from immunosurveillance to tumor escape. Nat. Immunol..

[30] Dunn G.P., Old L.J., Schreiber R.D. (2004). The three Es of cancer immunoediting. Annu. Rev. Immunol..

[31] Spranger S., Luke J.J., Bao R., Zha Y., Hernandez K.M., Li Y., Gajewski A.P., Andrade J., Gajewski T.F. (2016). Density of immunogenic antigens does not explain the presence or absence of the T-cell–inflamed tumor microenvironment in melanoma. Proc. Natl. Acad. Sci. USA.

[32] Ho W.W., Gomes-Santos I.L., Aoki S., Datta M., Kawaguchi K., Talele N.P., Roberge S., Ren J., Liu H., Chen I.X. (2021). Dendritic cell paucity in mismatch repair–proficient colorectal cancer liver metastases limits immune checkpoint blockade efficacy. Proc. Natl. Acad. Sci. USA.

[33] Tvedt T.H.A., Vo A.K., Ø B., Reikvam H. (2021). Cytokine Release Syndrome in the Immunotherapy of Hematological Malignancies: The Biology behind and Possible Clinical Consequences. *J*. *Clin*. *Med*. Nov.

[34] Abboud R., Wan F., Mariotti J., Arango M., Castagna L., Romee R., Hamadani M., Chhabra S. (2021). Cytokine release syndrome after haploidentical hematopoietic cell transplantation: an international multicenter analysis. Bone Marrow Transpl..

[35] Marciscano A.E., Anandasabapathy N. (2021/02/01/2021). The role of dendritic cells in cancer and anti-tumor immunity. Semin. Immunol..

[36] Buonaguro L., Petrizzo A., Tornesello M.L., Buonaguro F.M. (2011). Translating Tumor Antigens into Cancer Vaccines. Clin. Vaccin. Immunol..

[37] Small E.J., Schellhammer P.F., Higano C.S., Redfern C.H., Nemunaitis J.J., Valone F.H., Verjee S.S., Jones L.A., Hershberg R.M. (Jul 1 2006). Placebo-controlled phase III trial of immunologic therapy with sipuleucel-T (APC8015) in patients with metastatic, asymptomatic hormone refractory prostate cancer. J. Clin. Oncol..

[38] Zhao X., Subramanian S. (2017). Intrinsic Resistance of Solid Tumors to Immune Checkpoint Blockade Therapy. Cancer Res..

[39] Morad G., Helmink B.A., Sharma P., Wargo J.A. (Oct 14 2021). Hallmarks of response, resistance, and toxicity to immune checkpoint blockade. Cell.

