# Supplementary material for: Allogeneic tumor cell-derived extracellular vesicles stimulate CD8 T cell response in colorectal cancer
Source: Mol Ther Oncolytics. 2023 Sep 16;31:100727. doi: 10.1016/j.omto.2023.100727 (PMC10562189; doi:10.1016/j.omto.2023.100727)
Supplement: Document S1. Figure S1 [file mmc1.pdf]

OMTO, Volume *31*

Supplemental information

Allogeneic tumor cell-derived extracellular vesicles stimulate CD8 T cell response in colorectal cancer

Travis J. Gates, Dechen Wangmo, Xianda Zhao, and Subbaya Subramanian

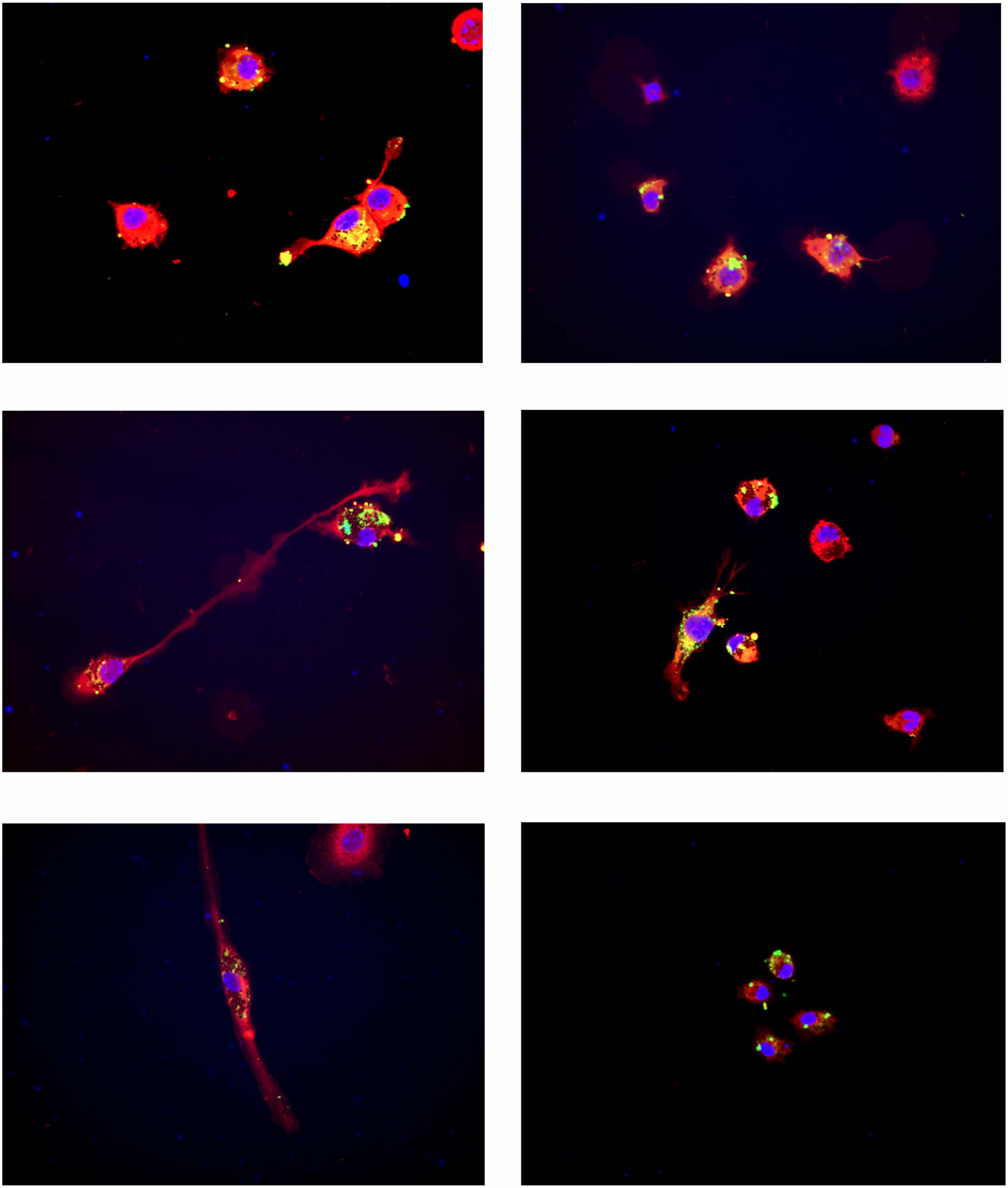

Figure S1: Fluorescence microscopy images demonstrating the uptake of labeled DiO TEVs (green) by DCs (red). Nuclei stained with DAPI (blue).
